# Impact of platelet transfusion on outcomes in trauma patients

**DOI:** 10.1186/s13054-022-03928-y

**Published:** 2022-02-21

**Authors:** S. R. Hamada, D. Garrigue, H. Nougue, A. Meyer, M. Boutonnet, E. Meaudre, A. Culver, E. Gaertner, G. Audibert, B. Vigué, J. Duranteau, A. Godier, and the TraumaBase Group, Paer-Selim Abback, Gérard Audibert, Tobias Gauss, Thomas Geeraerts, Anatole Harrois, Olivier Langeron, Marc Leone, Julien Pottecher, Laurent Stecken, Jean-Luc Hanouz

**Affiliations:** 1 Service d’Anesthésie Réanimation Hôpital Européen Georges Pompidou APHP, Université de Paris, and CESP, INSERM U 10-18, Université Paris-Saclay, 20 Rue Leblanc, Hôpital Européen Georges Pompidou, 75015 Paris, France; 2 CHU de Lille, Pôle d’Anesthésie Réanimation, 59000 Lille, France; 3 AP-HP, Service d’Anesthésie Réanimation, HEGP, Paris, France; 4 Département d’Anesthésie Réanimation, CHU de Strasbourg Hautepierre, Strasbourg, France; 5 Département d’Anesthésie Réanimation, HIA Percy, Clamart, France; 6 Département d’Anesthésie Réanimation, HIA Saint Anne, Toulon, France; 7 Département d’Anesthésie Réanimation, Hôpital Nord, Marseille, France; 8 Département d’Anesthésie Réanimation, Hôpital de Colmar, Colmar, France; 9 Département d’Anesthésie Réanimation, Hôpital de Nancy, Nancy, France; 10 AP-HP, Département d’Anesthésie Réanimation, Hôpital du Kremlin Bicêtre, Le Kremlin-Bicêtre, France; 11 Service d’Anesthésie Réanimation Hôpital Européen Georges Pompidou APHP, INSERM UMRS-1140, Université de Paris, Paris, France

**Keywords:** Trauma, Haemorrhage, Platelet count, Platelet transfusion, Outcome

## Abstract

**Background:**

Trauma-induced coagulopathy includes thrombocytopenia and platelet dysfunction that impact patient outcome. Nevertheless, the role of platelet transfusion remains poorly defined. The aim of the study was 1/ to evaluate the impact of early platelet transfusion on 24-h all-cause mortality and 2/ to describe platelet count at admission (PCA) and its relationship with trauma severity and outcome.

**Methods:**

Observational study carried out on a multicentre prospective trauma registry. All adult trauma patients directly admitted in participating trauma centres between May 2011 and June 2019 were included. Severe haemorrhage was defined as ≥ 4 red blood cell units within 6 h and/or death from exsanguination. The impact of PCA and early platelet transfusion (i.e. within the first 6 h) on 24-h all-cause mortality was assessed using uni- and multivariate logistic regression.

**Results:**

Among the 19,596 included patients, PCA (229 G/L [189,271]) was associated with coagulopathy, traumatic burden, shock and bleeding severity. In a logistic regression model, 24-h all-cause mortality increased by 37% for every 50 G/L decrease in platelet count (OR 0.63 95% CI 0.57–0.70; *p* < 0.001). Regarding patients with severe hemorrhage, platelets were transfused early for 36% of patients. Early platelet transfusion was associated with a decrease in 24-h all-cause mortality (versus no or late platelets): OR 0.52 (95% CI 0.34–0.79; *p* < 0.05).

**Conclusions:**

PCA, although mainly in normal range, was associated with trauma severity and coagulopathy and was predictive of bleeding intensity and outcome. Early platelet transfusion within 6 h was associated with a decrease in mortality in patients with severe hemorrhage. Future studies are needed to determine which doses of platelet transfusion will improve outcomes after major trauma.

**Supplementary Information:**

The online version contains supplementary material available at 10.1186/s13054-022-03928-y.

## Background

Acute post-traumatic haemorrhage is the first cause of preventable death [1, 2]. Deaths from haemorrhage occur early, typically within the first 6 h. Trauma-induced coagulopathy (TIC) is a complex, multifactorial failure of haemostasis that occurs in 25% of severely injured patients and results in a fourfold higher mortality [3]. It appears immediately after trauma, at the scene of the accident, before any treatment [4]. It is characterized by hyperfibrinolysis, hypofibrinogenemia, systemic anticoagulation and endothelial dysfunction. It is secondarily aggravated by shock, hypothermia, acidosis, hypocalcaemia and dilution with filling solutions. More recently, the role of platelets in TIC has been highlighted, including thrombocytopenia, often mild [5] and especially platelet dysfunction [6]. Both quantitative and qualitative platelet damages are all the more marked when tissue injury is severe, transfusion is large, and hypoperfusion is intense and prolonged [7]. It is also associated with mortality. Nevertheless, the pathophysiological role of platelets in TIC remains poorly understood and is still an area of intense research.

The guidelines for the management of major bleeding and coagulopathy following trauma specify that platelet transfusion should maintain a platelet count above 50 G/L, or even above 100 G/L in the event of active bleeding or brain injury [8]. However, improved knowledge on trauma-induced platelet dysfunction and its prognostic role has led to the hypothesis that earlier and more aggressive platelet transfusion would improve the outcome of bleeding severe trauma patients. Observational studies on severe trauma patients support this hypothesis: they show an association between early platelet transfusion and mortality [9] but also between the ratio of platelet units to packed red blood cell units (RBCs) and mortality [10, 11]. The substudy of the PROPPR randomized trial showed that platelet transfusion was associated with improved haemostasis and reduced mortality in severely injured, bleeding patient [12]. Conversely, other observational data suggest that platelet transfusion may be inefficient [13], or may even paradoxically worsen platelet function [14]. However, no randomized trial has evaluated the potential benefit of early platelet transfusion in traumatic haemorrhagic shock. So, lacking high level of evidence on the subject, the role of platelet transfusion in severe trauma management remains poorly defined and recommendations are limited to expert opinions.

The aim of this study was to evaluate the impact of early platelet transfusion on 24-h all-cause mortality. Secondary objective was to analyse modalities of platelet transfusion and to describe platelet count at admission and its relationship with trauma severity and outcome.

## Methods

This is a retrospective study conducted on patients whose data were prospectively collected in the Traumabase® between January 2012 and June 2019. The Traumabase® (www.traumabase.eu) is a French major trauma registry initiated in 2010. Patients included in the Traumabase® are suspected of severe trauma from the scene and admitted (primarily or secondarily) to the participating trauma centres (*n* = 14). The Traumabase® is in accordance with all requirements from the Advisory Committee for the processing of research information in the field of health (CCTIRS), the French National Commission on Computing and Liberty (CNIL, authorization number 911461) and meets the requirements of the local and national ethics committee (Comité de Protection des Personnes, Paris VI). The structure of the database integrates algorithms for consistency and coherence, and the data monitoring is performed by a central administrator [15].

We included all trauma patients collected in the registry during the predefined period. Patients managed after secondary transfer, aged < 15 years and those receiving antiplatelet agents were excluded from analysis. Two subpopulations were studied, referred as *bleeding severity*: (i) trauma patients presenting severe haemorrhage (i.e. transfusion of at least 4 RBCs within the first 6 h of management and/or death from haemorrhagic causes whatever the level of transfusion); (ii) patients with massive transfusion (i.e. transfusion of at least 10 RBCs within the first 24 h).

The course of trauma patients was structured in five stages: prehospital, resuscitation room (RR), first 6 h (H6), first 24 h (H24), and intensive care unit length of stay (ICU LOS). Collected data regarding transfusion included the number of RBCs, fresh frozen plasma (FFP) and platelet units (PUs) in RR, H6, H24 and ICU LOS. For the study purpose, we considered that one platelet concentrate was equivalent to five donor PUs. The transfusion ratios, FFP:RBC and PU:RBC were calculated. In cases where the ratio could not be obtained in the mutually exclusive cases of a numerator or denominator equal to 0, a dummy value of 0.5 was imputed to preserve the observation in the description and analysis. Of note, all participating centres followed the European guidelines on the management of major bleeding and coagulopathy following trauma, and all-centre massive transfusion protocols contained platelets every 3-to-6 RBC. Severity scores such as Simplified Acute Physiology Score (SAPS2), Injury Severity Score (ISS) and Sequential Organ Failure Assessment (SOFA) were calculated from the values of the first 24 h and recorded in the database. The 24-h and ICU all-cause mortality were used as the final outcomes.

Patients were categorized according to admission platelet count into one of the following categories: 0–49 G/L, 50–99 G/L, 100–149 G/L, 150–299 G/L and ≥ 300 G/L. Patients were further categorized into four injury severity groups according to Injury Severity Score (ISS) and admission base deficit: (1) ISS ≥ 25, base deficit ≥ −6; (2) ISS ≥ 25, base deficit < −6; (3) ISS < 25, base deficit ≥ −6; and (4) ISS < 25, base deficit < -6. These cut-offs for ISS and base deficit have been used in prior studies as reliable markers for anatomical injury burden (ISS) and shock severity (base deficit) [5].

The primary objective was to assess the association between early platelet transfusion (defined as platelet transfusion within the first 6 h) and 24-h all-cause mortality.

Secondary objectives targeted the correlation between platelet count and trauma severity (injury, coagulopathy and acidosis), the association between platelet count and outcome, including bleeding intensity and mortality (24-h all-cause and ICU mortality), the association between platelet transfusion and blood product consumption.

### Statistical methods

The report follows the Strobe guidelines [16]. Categorical variables are presented as counts and percentages (%) and 95% confidence interval (95%CI, when appropriate), while continuous variables are expressed as mean (standard deviation) or median [quartile 1, 3] according to their distribution. Multimodal plotting was used to represent associations between clinical characteristics and platelet count and between transfusion modalities and time.

To assess whether early platelet transfusion was an independent risk factor of 24-h all-cause mortality, we performed first bivariate analyses using Chi-square test and Student’s t test (or Mann–Whitney test) depending on variable type, to assess crude associations and to identify confounders (i.e. covariates statistically associated with 24-h all-cause mortality and those associated with early platelet transfusion). Then, multivariate analysis was performed after imputation of missing values (all were < 20% missing) to identify independent predictors of 24-h all-cause mortality. The variables having a *p* value < 0.20 in the bivariate analysis were checked for collinearity and then entered in the logistic regression model, as well as well-established predictors of mortality, previously listed by a group of experts on a Delphi [17]. Calibration and discrimination of the models were computed. Statistical analysis was performed with R software version 3.3.3 for Windows (R Foundation for Statistical Computing, Vienna, Austria).

## Results

**Fig. 1:**
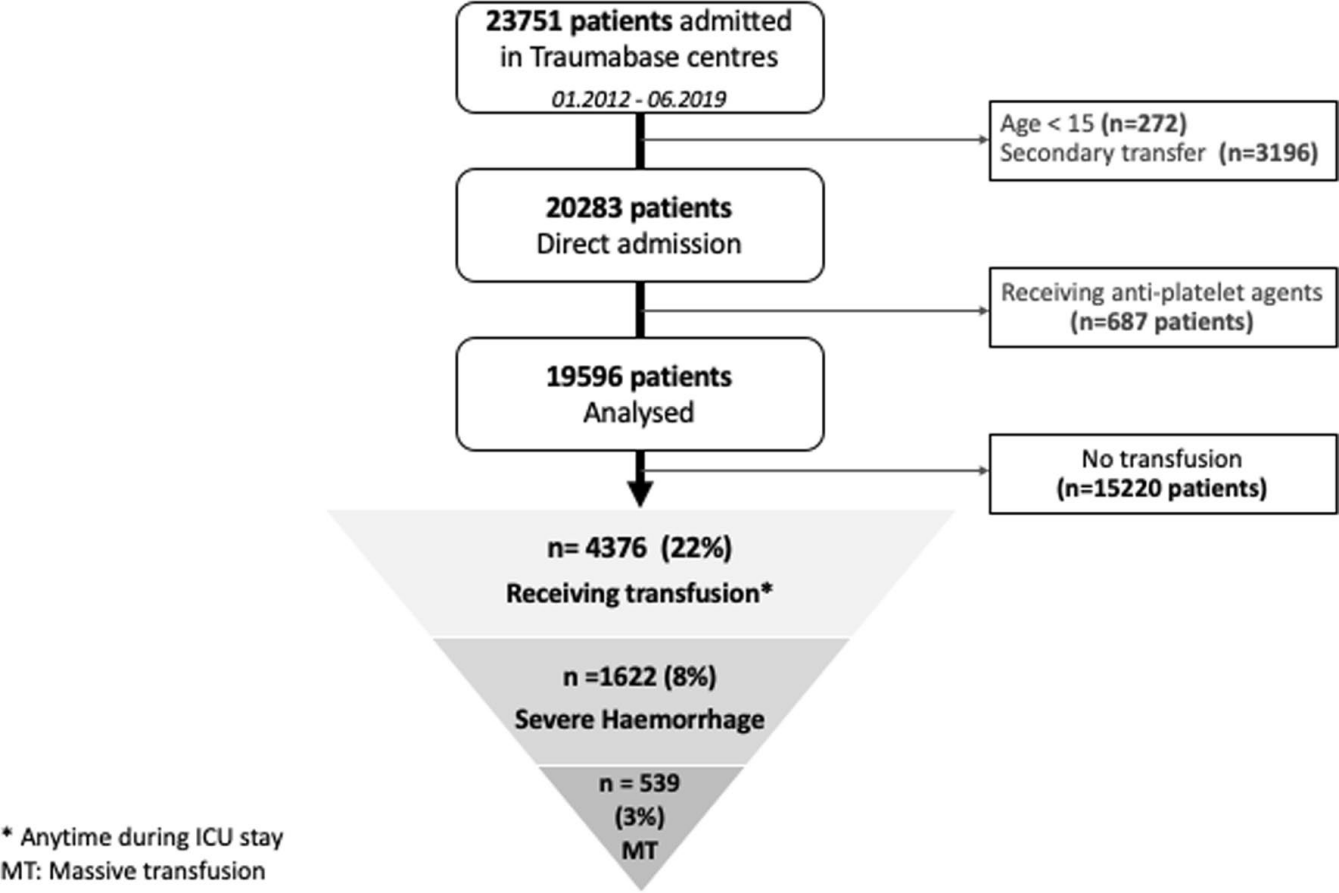
Flowchart of the study

During the study period, 23,751 patients were admitted in the participating trauma centres. After exclusion of patients out of defined criteria, 19,596 remained for analysis, 1622 (8%) presented with severe haemorrhage and 539 (3%) received massive transfusion (flowchart in Fig. 1). The characteristics of each subpopulation are presented in Table 1.

**Table 1:**
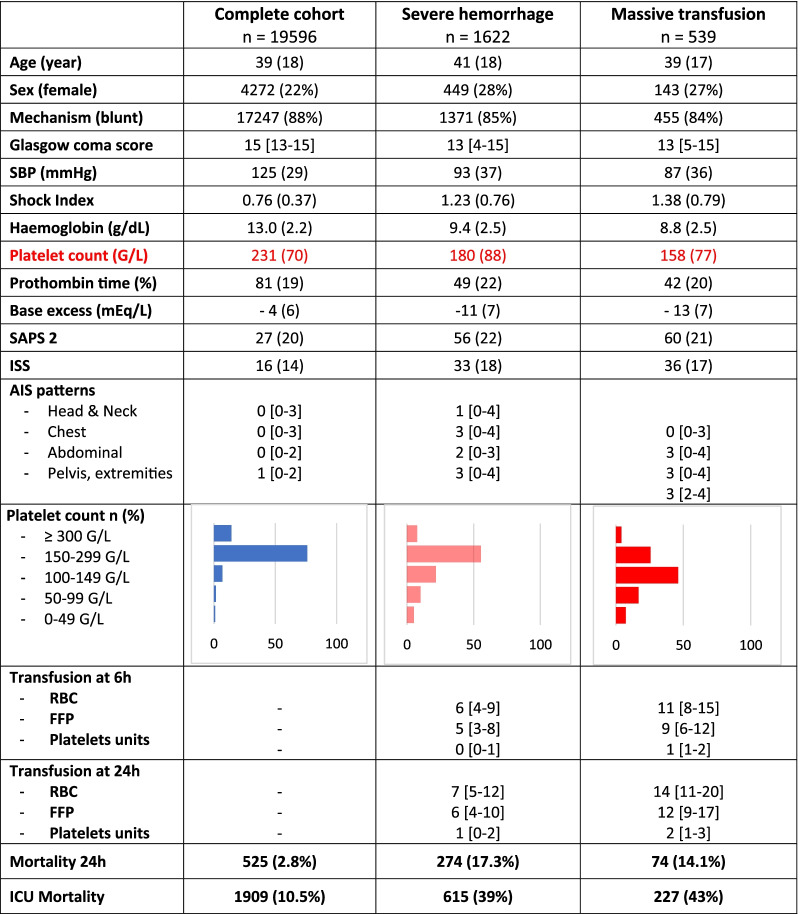
Characteristics of each subpopulation

Severe Hemorrhage: transfusion of at least 4 RBCs within the first 6 h of management and/or death from haemorrhagic causes without having reached the 4 transfused RBCs

Massive transfusion: transfusion of at least 10 RBCs within the first 24 h

Data are presented as mean (SD) for normally distributed data and median [Quartile 1, 3] for nonparametric data

*AIS* Abbreviated Injury Score, *ISS* Injury Severity Score, *FFP* fresh-frozen plasma, *RBC* red blood cells, *PU* platelet unit, *SBP* systolic arterial pressure, *SAPS* Simplified Acute Physiology Score

### Platelet count and severity

**Fig. 2:**
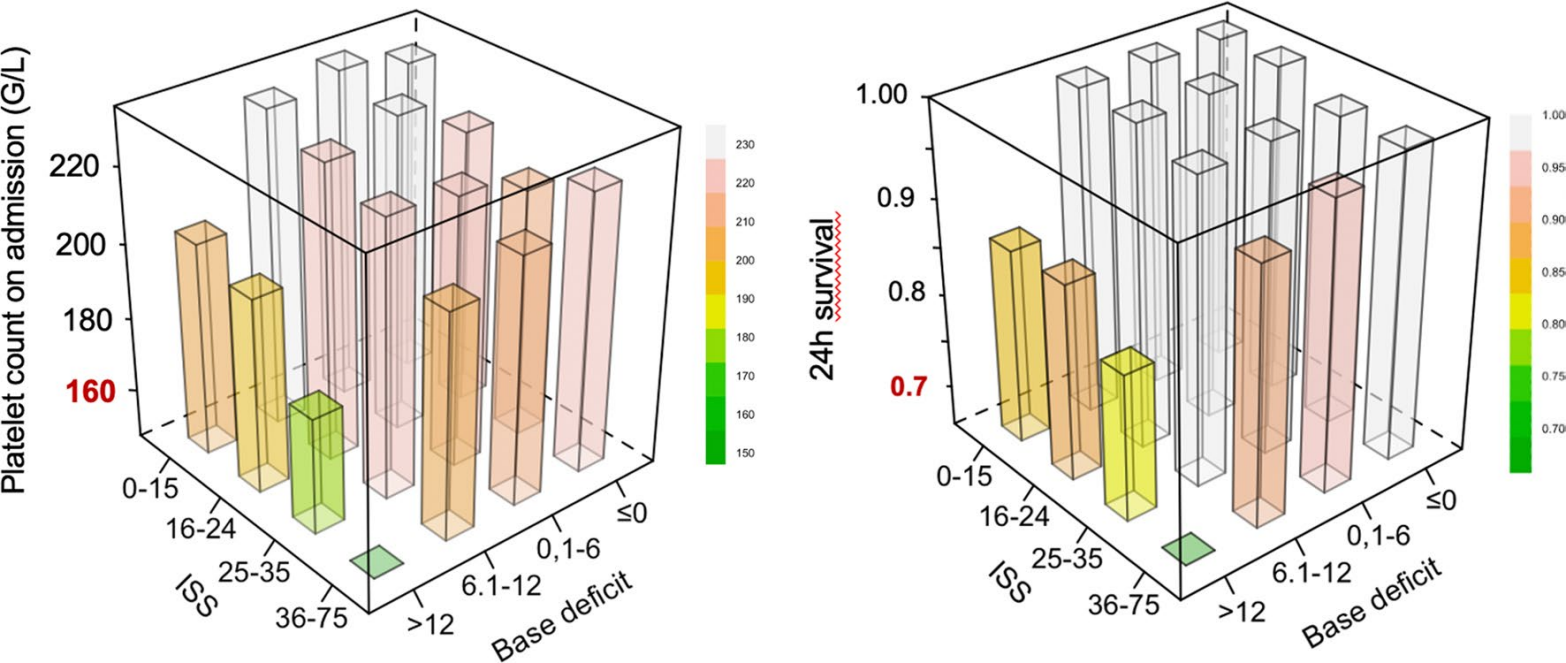
Relationship between traumatic burden and intensity of shock (i.e. Injury Severity Score (ISS) and base deficit (BD). A/ Multivariate plot of median platelet count against both ISS and BD. The worst platelet counts were seen in patients admitted with the combination of the most severe injuries and highest BD (median Platelet count = 146 G/L for patients having ISS > 35 and BD > 12; with *p* < 0.05 compared with all other groups). B/ Multivariate plot of 24-h all-cause survival rate against both ISS and BD. The worst survival rates were seen in patients admitted with the combination of the most severe injuries and highest BD (66% for patients having ISS > 35 and BD > 12; with *p* < 0.05 compared with all other groups)

Platelet count at admission varied from 2 G/L to 979 G/L, with a median of 229 G/L [189, 271]. Platelet count was associated wi*t*h acute trauma coagulopathy as it was correlated with prothrombin time ratio (*r* = 0.31, *p* < 0.001). The lower the admission platelet count, the more coagulopathic the patients (prothrombin time ratio > 1.5): 18% were coagulopathic when platelet count was above 150 G/L, 52% were coagulopathic when between 100 and 150 G/L, and 67% when below 100 G/L. Platelet count was also weakly correlated with fibrinogen concentration (*r* = 0.27, *p* < 0.001). Moreover, platelet count was associated with the main drivers of coagulopathy, i.e. with ISS (as a proxy for anatomical injury and tissue damage) and BD (as a proxy for shock severity)(Fig. 2): the worst platelet counts were measured in patients admitted with the combination of the most severe injuries (ISS > 35) and highest base deficit (BD > 12).

**Fig. 3:**
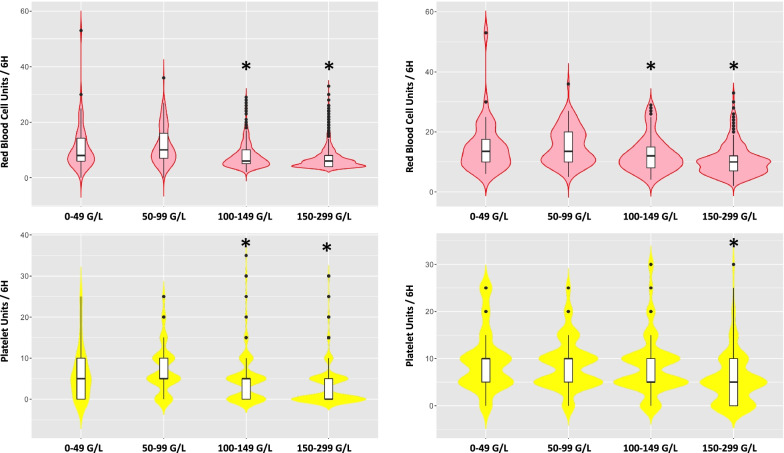
Transfusion of red blood cell units (in red) and platelet units (in yellow) at 6 h according to platelet count at admission in patients with severe haemorrhage (left) and patients with massive transfusion (right). Violin plots represent both distribution and density of data: they visually combine the plotting of descriptive data of the box-and-whisker plots (median, interquartile ranges, minimum and maximum) with the addition of a rotated kernel density plot on each side. The violin plots show the presence of different peaks, at different positions reflecting different transfusion behaviours. The x-axis presents admission platelet count categories. Transfusion requirements according to admission platelet count were compared (t-tests): *p < 0.05: transfusion requirement of the concerned admission platelet count category significantly different from the immediate previous category

Platelet count at admission predicted bleeding severity (SH or MT): counts were lower in patients with severe haemorrhage or massive transfusion compared to the complete cohort (180 (88) G/L and 158 (77) G/L vs 231 (70) G/L, respectively, *p* < 0.01) (Table 1). Moreover, platelet count was correlated with haemoglobin concentration at admission (r = 0.24, p < 0.001) and with RBC requirements over 24 h: for every 50 G/L decrease in platelet count, one more unit of RBC was transfused (1, 95% CI 0.8–1.2, *p* < 10^–3^). Violin plots presented in Fig. 3 show that both RBC transfusion and platelet transfusion increased when admission platelet count decreased.

**Fig. 4:**
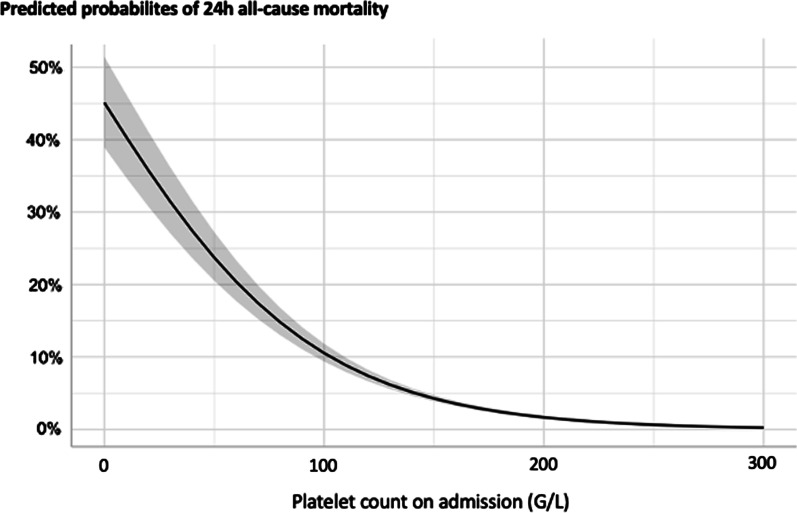
Predicted probability of 24-h all-cause mortality according to platelet count on admission

Platelet count at admission was associated with mortality (Fig. 4). In the whole cohort, univariate logistic regression showed that, for every 50 G/L decrease in admission platelet count, the odds of 24-h all-cause mortality increased by 61% (OR 0.39, 95% CI 0.36–0.42, *p* < 10^–3^) and ICU mortality by 44% (OR 0.56, 95% CI 0.54–0.58, *p* < 10^–3^). Moreover, in a logistic regression model predicting 24-h all-cause mortality and adjusted on universally admitted confounders (ISS, Glasgow Coma Score and base deficit)[5], the odds of death increased by 37% for every 50 G/L decrease in platelet count (OR 0.63, 95% CI 0.57–0.70, *p* < 0.001) (Additional file 1: Table S1).

### Platelets and transfusion

Platelet transfusion requirements during the ICU stay varied according to bleeding severity: platelets were transfused to 1335 patients of the whole cohort (7%), to 901 patients of those with severe haemorrhage (56%) and to 470 of the massively transfused patients (87%).

Similarly, platelet transfusion requirements varied according to admission platelet count. Among 233 patients presenting with minimal platelet count < 50 G/L, 159 (68%) received platelet transfusion within the first 6 h and 74 (32%) did not. Among those latter, 22 (30%) died within 24 h, and half of them during initial resuscitation. Among patients receiving early platelet transfusion, 33 (21%) died within 24 h (*n* = 5 during initial resuscitation).

Regarding patients with SH, 640 (40%) did not receive platelet transfusion within the first day (4% missing data), 176 patients (11%) received platelet transfusion in the resuscitation room, 590 patients (36%) received platelets thereafter but within the first 6 h and 148 (9%) patients within the following 18 h. This resulted in low platelet:plasma ratios (0.5 [0.1; 1]) and low platelet:RBC (0.3 [0.1; 0.7]) ratios within the first six hours (Fig. 3, Additional file 3: Fig. S1). Patients who received platelet transfusion within 6 h were more severely injured, had more severe shock and coagulopathy at admission with lower prothrombin time, lower fibrinogen concentration and lower platelet count than those who did not (Table 2, *p* < 0.001). They also required more RBC transfusion (*p* < 0.001).

**Table 2:** Patient characteristics according to early platelet transfusion or not in the Severe Hemorrhage sub-population (bivariate analysis)

	Early platelet transfusion (*n* = 766)	No early platelet transfusion (*n* = 767)	*p*
Age, years	40 (17)	41 (19)	0.07
Sex, female	205 (25%)	225 (29%)	0.22
Shock Index (prehosp)	1.6 (0.74)	1.4 (0.57)	< 0.001
SAP, mmHg	90 (37)	100 (34)	< 0.001
FC, bpm	103 (34)	101 (31)	0.18
GCS	12 [3–15]	14 [6–15]	< 0.001
Base deficit	12.3 (7.3)	9.5 (6.4)	< 0.001
Haemoglobin (g/dL)	9.0 (2.5)	10.0 (2.3)	< 0.001
Prothrombin time (%)	44 (21)	56 (20)	< 0.001
Fibrinogen, g/L	1.3 (0.8)	1.6 (0.8)	< 0.001
Platelets, G/L	161 (84)	202 (82)	< 0.001
RBC H6	8 [6–13]	5 [4–6]	< 0.001
FFP H6	8 [5–11]	4 [4–5]	< 0.001
PCU H6	5 [5–10]	0 [0–0]	< 0.001
SAPS 2	59 (21)	51 (22)	< 0.001
SOFA	11 (4)	9 (4)	< 0.001
ISS	34 [24–48]	29 [17–41]	< 0.001
AIS head	0 [0–4]	2 [0–3]	0.83
AIS thorax	3 [0–4]	3 [0–4]	0.001
AIS abdomen	2.5 [0–4]	2 [0–3]	< 0.001
AIS pelvis	3 [0–4]	3 [1–4]	0.47
Mortality 24 h	116 (16%)	102 (14%)	< 0.001
ICU Mortality	316 (43%)	231 (31%)	< 0.001
Cause of death			
Central nervous system	71 (22%)	69 (30%)	
Exsanguination	107 (34%)	69 (30%)	< 0.001
Multiple organ failure	94 (30%)	43 (19%)	
Withdrawal	29 (9%)	22 (9%)	
Septic shock	0 (0%)	6 (3%)	
Other	15 (5%)	22 (9%)	

1533 patients having data for platelet transfusion over 1622 patients in the cohort

Data are presented as mean (SD) for normally distributed data and median [quartile 1, 3] for nonparametric data

*AIS* Abbreviated Injury Score, *GCS* Glasgow Coma Score, *HR *maximal heart rate, *ISS* Injury Severity Score, *FFP* fresh-frozen plasma, *RBC* red blood cells, *PCU* platelet concentrate units, *SAPS* Simplified Acute Physiology Score, *SOFA* Sequential Organ Failure Assessment

Multivariate logistic regression analysis showed that early platelet transfusion (within 6 h) was an independent protective factor for 24-h all-cause mortality: OR 0.56, 95% CI 0.38–0.84, *p* = 0.004) in the SH subpopulation (Table 3). The same results were observed in the MT subgroup, including patients dying from haemorrhage without obtaining 10 RBC units in order to account for a potential survival bias: OR 0.33, 95% CI 0.20–0.55, *p* = 0.007) (Additional file 2: Table S2).

**Table 3:** Multivariate predictors of 24-h all-cause mortality in trauma patients presenting severe haemorrhage

	Odds ratio [2.5–97.5%]
Intercept	0.86 [0.17–4.33]
Early platelet tranfusion*	0.56 [0.38–0.84]
Age*	1.02 [1.01–1.03]
Sex (m)	1.42 [0.92–2.21]
ASA 1	0.76 [0.48–1.20]
Motor GCS*	0.88 [0.79–0.99]
Mydriasis	1.24 [0.68–2.25]
Cardiac arrest*	2.10 [1.32–3.33]
Shock index	1.11 [0.78–1.58]
Norepinephrin use	1.07 [0.67–1.70]
Base Deficit*	1.09 [1.06–1.13]
Haemoglobin	1.01 [0.93–1.10]
Prothrombin time*	0.96 [0.94–0.97]
Fibrinogen*	0.56 [0.35 -0.87]
Ratio (FFP:RBC)*	0.20 [0.11–0.35]
Tranexamic acid	0.81 [0.44–1.51]
AIS head (≥ 3)*	1.67 [1.07–2.65]
ISS*	1.02 [1.01–1.03]

The logistic regression model was adjusted on well-established predictors of mortality, previously listed by a group of experts on a Delphi [17] and on confounders of early platelet transfusions identified on bivariate analysis (*p* < 0.2). Early platelet transfusion was defined as platelet transfusion within the first 6 h. Odds ratios with 95% confidence intervals [OR (95% CI)]

Akaike criteria 810

Calibration: Hosmer–Lemeshow test *p* = 0.117

Discrimination: AUC 0.93, 95% CI (0.91–0.94)

*p < 0.05

## Discussion

This analysis of 19,596 trauma patients showed that platelet count at admission was a biomarker of trauma severity and was predictive of outcome, including bleeding intensity and mortality. Early platelet transfusion within 6 h was associated with a lower 24-h mortality.

So far, the role of platelets in trauma-induced coagulopathy remains unclear. Platelets play a central role in haemostasis, as an essential substrate of clot formation. Immediately after trauma, whereas coagulation factors, including fibrinogen, decrease and fibrinolysis, is activated, platelet count remains surprisingly essentially normal and the prevalence of thrombocytopenia at hospital admission is usually low. The conservation of rather normal counts results from a post-traumatic enhanced and compensatory release of platelets, from their storage pool in the spleen and the bone marrow [18]. As a result, admission platelet count is > 100 G/L in most trauma patients, even in those massively transfused [5, 12]. Nevertheless, variations of platelet count within “normal ranges” reflect trauma severity (Fig. 3D). Several observational studies confirmed that admission platelet count was a biomarker of trauma severity as it was associated with injury severity, shock intensity and with coagulopathy at admission [5].

Admission platelet count is also a prognostic marker. In our study, platelet count predicted bleeding severity and transfusion requirements (Fig. 3 violin plots). Similarly, in a cohort of massively transfused trauma patients, patients with a higher admission platelet count receive fewer RBCs: for every 50 G/L increase in platelet count, patients received 0.7 fewer units of blood within the first 6 h and one less unit of blood within the first 24 h (5). Several studies also reported that admission platelet count was associated with mortality. Hess et *al.* found a stepwise relationship between platelet count and in-hospital mortality for nine levels of injury severity [19]. Similarly, Brown et *al.* showed that the probability of death at 24 h decreased as the admission platelet count increased [5]. Nevertheless, as platelet count is closely related to trauma severity and coagulopathy that are well-established predictors of mortality, we performed a multivariate logistic regression model to assess the specific role of platelet count on outcome. Our study demonstrated for the first time that every 50 G/L decrease of admission platelet count was an independent predictor of early mortality (OR 0.39).

Subsequently, we hypothesized that early platelet transfusion would improve both platelet count and trauma-induced platelet dysfunction, thereby leading to a decreased mortality. Despite a strong rationale, the efficacy of platelet transfusion in trauma patients remains controversial. There is no large, randomized trial to support the benefit of platelet transfusion on patient outcome, and available data lead to conflicting conclusions. The substudy of the PROPPR trial showed that early platelet transfusion was associated with improved haemostasis and reduced mortality but was limited to severely bleeding trauma patients [12]. On the opposite, Sambasivan and colleagues reported that high ratios of platelet unit:RBC (> 1:2) administrated to non-massively transfused trauma patients had no effect on mortality compared to low ratios (≤ 1:2) but significantly decreased ventilator-free and ICU-free days [20]. In another study also including non-massively transfused blunt injury victims, platelet transfusion was found to increase the risk of acute respiratory distress syndrome without any survival benefit, but platelet transfusion was recorded over 48 h and only 488 patients were included [21]. Our study provided the opportunity to examine a large cohort of trauma patients and suggested that early platelet transfusion improved survival, not only in massively transfused patients but also in severely bleeding trauma patients. The other variables that were significant predictors of mortality were previously known and thus not discussed here [17].

The current *European Guidelines on the management of major bleeding and coagulopathy following trauma* recommend maintaining a platelet count greater than 50 G/L for patients with ongoing bleeding, or greater than 100 G/L if associated with traumatic brain injury. Nevertheless, such thresholds may be insufficient after severe trauma. First, platelet count, although essentially higher than 100 G/L, is a prognostic marker following trauma. Early platelet transfusion improved survival, when 96% of early platelet transfusion were performed to patients with an admission platelet count > 100 G/L. Similarly, in the PROPRR substudy that also supports transfusion of platelets as early as possible, only 2% of patients had platelet count below 100 G/L [12]. These data support platelet transfusion despite normal platelet count. Second, low thresholds expose to dilution: in the case of massive RBC and FFP transfusion without platelet transfusion, platelet count will decrease during the course of trauma resuscitation. In a model of major surgery, a blood loss of one blood volume replaced with RBC and colloids led to decreased platelet count by half, with a large interindividual variability [22, 23]. A decrease in the platelet count to 50 G/L is expected when RBC equivalent to two blood volumes has been transfused (ref). Third, the quality of evidence that supports 50 G/L as an haemostatic threshold in trauma is very low and was defined from small series of surgical patients, with isolated thrombocytopenia and not from trauma patients with complex haemostatic disorders [24, 25]. Fourth, these thresholds do not take into account trauma-induced platelet dysfunction. Platelet dysfunction has been identified as one of the mechanisms of trauma-induced coagulopathy and deeply impacts platelet aggregation and activation pathways. Platelet dysfunction occurs in approximately 50% of trauma patients and is associated with increased morbidity and mortality. This is an additional argument to increase platelet transfusion threshold [3].

This study presents some limitations. First, it is a retrospective analysis, impairing the collection of some specific confounders such as local situation or patient dynamic clinical response to treatments. Nevertheless, data collection was prospective, and the use of the Traumabase® registry secures data processing, limits missing data through responsive data-monitoring and integrates control of biases inherent to retrospective data collection [26]. Moreover, the logistic regression model that assessed the relationship between platelet transfusion and mortality was performed rigorously in order to control for confounding factors. Second, some degree of survival bias cannot be ruled out when considering platelet transfusion within 6 h of hospital admission. Third, the exact dose of transfused platelet units is unknown as the label of the recorded variable used was “platelet concentrates”. Fourth, we cannot rule out a part of a survival bias as the time period of 6 h to define early platelet transfusion is long. Nevertheless, too few patients were transfused during initial resuscitation to allow proper analysis on this subpopulation. Last, our study did not catch platelet dysfunction and thus is unable to integrate this element to explore its relationship with poor outcomes.

## Conclusion

Admission platelet count, although mainly in normal range, was associated with trauma severity and coagulopathy, and was predictive of bleeding intensity and outcome. Early platelet transfusion within 6 h was associated with a decrease in mortality. A normal platelet count might be an insufficient goal after severe trauma, and severe trauma patients might benefit from an earlier and higher threshold for platelet transfusion. Future studies are needed to determine which doses of platelet transfusion will improve outcomes after major trauma.

## Supplementary Information

**Additional file 1**. **Table S1**: Multivariate logistic regression analysis: predictors of 24-h all-cause mortality (on complete cases from the whole cohort, *n* = 12278). All p values < 10^−6^

**Additional file 2**. **Table S2**: Multivariate analysis of 24-hour all-cause mortality in the MT subpopulation

**Additional file 3**.** Fig. S1**: Cumulative transfusions of blood products (RBC, FFP, PU) during the trauma patient clinical pathway until ICU discharge

## Data Availability

Data are from the Traumabase, a collaborative project that consists of trauma practitioners, from different centres all over the country, sharing the registry for research and public health issues. Even though datasets are de-identified, the National Commission for data protection (CNIL) has imposed restrictions on data sharing since they contain sensitive informations. Conventions are signed for researcher before any access to the data. For data access, interested researchers can contact the Traumabase scientific committee on reasonable request with the following email address: contact@traumabase. eu (secretary of the scientific committee- Dr. Anatole Harrois).

## Abbreviations

BD: Base deficit
CI: Confidence interval
FFP: Fresh-frozen plasma
ICU: Intensive care unit
ISS: Injury Severity Score
LOS: Length of stay
MT: Massive transfusion
OR: Odds ratio
PU: Platelet units
RBC: Packed red blood cell units
SAPS 2: Simplified Acute Physiology Score
SH: Severe hemorrhage
SM: Supplementary material
SOFA: Sequential Organ Failure Assessment
TIC: Trauma-induced coagulopathy
