# Supplementary material for: Impact of platelet transfusion on outcomes in trauma patients
Source: Crit Care. 2022 Feb 21;26:49. doi: 10.1186/s13054-022-03928-y (PMC8862339; doi:10.1186/s13054-022-03928-y)
Supplement: Supplementary file 1 — Additional file 1. Table S1: Multivariate logistic regression analysis: predictors of 24-h all-cause mortality (on complete cases from the whole cohort, n = 12278). All p values < 10−6 [file 13054_2022_3928_MOESM1_ESM.docx]

Table SM1. Multivariate logistic regression analysis: predictors of 24-hour all-cause mortality (on complete cases from the whole cohort, n=12278). All p values < 10^-6^

|  | **Odds Ratio [2.5%-97.5%]** |
| --- | --- |
| **Intercept** | 0.1 [0.06-0.19] |
| **Platelets per 50 G/L** | 0.63 [0.57-0.70] |
| **ISS** | 1.03 [1.02-1.04] |
| **Glasgow Coma Scale** | 0.85 [0.82-0.88] |
| **Base Deficit** | 1.14 [1.11-1.16] |

Area Under The Receiver Curve (AUC) : 0.94 []
