# Supplementary material for: Impact of platelet transfusion on outcomes in trauma patients
Source: Crit Care. 2022 Feb 21;26:49. doi: 10.1186/s13054-022-03928-y (PMC8862339; doi:10.1186/s13054-022-03928-y)
Supplement: Supplementary file 2 — Additional file 2. Table S2: Multivariate analysis of 24-hour all-cause mortality in the MT subpopulation [file 13054_2022_3928_MOESM2_ESM.docx]

**Table Supplementary Material 2**: Multivariate analysis of mortality in the MT sub-population

|  | **Odds Ratio [2.5%-97.5%]** |
| --- | --- |
| **Intercept** | 3.17 [0.46-22.7] |
| **Early platelet Tranfusion *** | 0.33 [0.20-0.55] |
| **Age *** | 1.01 [0.99-1.02] |
| **Sexe (m)** | 1.26 [0.76-2.12] |
| **Base Deficit *** | 1.08 [1.04-1.12] |
| **ASA 1** | 0.65 [0.37-1.13] |
| **Motor GCS *** | 0.89 [0.78-1.02] |
| **Mydriasis** | 0.96 [0.43-2.13] |
| **Cardiac arrest *** | 2.55 [1.48-4.41] |
| **Shock Index** | 0.95 [0.64-1.40] |
| **Norepinephrin** | 1.00 [0.58-1.72] |
| **Hemoglobin** | 1.02 [0.92-1.13] |
| **Prothrombin time *** | 0.97 [0.95-0.99] |
| **Fibrinogen*** | 0.89 [0.53 -1.37] |
| **Ratio (FFP:RBC) *** | 0.14 [0.06-0.30] |
| **Tranexamic acid** | 0.84 [0.38-1.88] |
| **AIS head (≥3) *** | 1.40 [0.83-2.38] |
| **ISS *** | 1.01 [0.83-2.38] |

*p<0.05
Akaike criteria 564

Calibration : Hosmer Lemeshow test p=0.25

Discrimination : AUC 0.88, _95%_ CI (0.85-0.91)
