# Supplementary figures and images for: Impact of platelet transfusion on outcomes in trauma patients
Source: Crit Care. 2022 Feb 21;26:49. doi: 10.1186/s13054-022-03928-y (PMC8862339; doi:10.1186/s13054-022-03928-y)

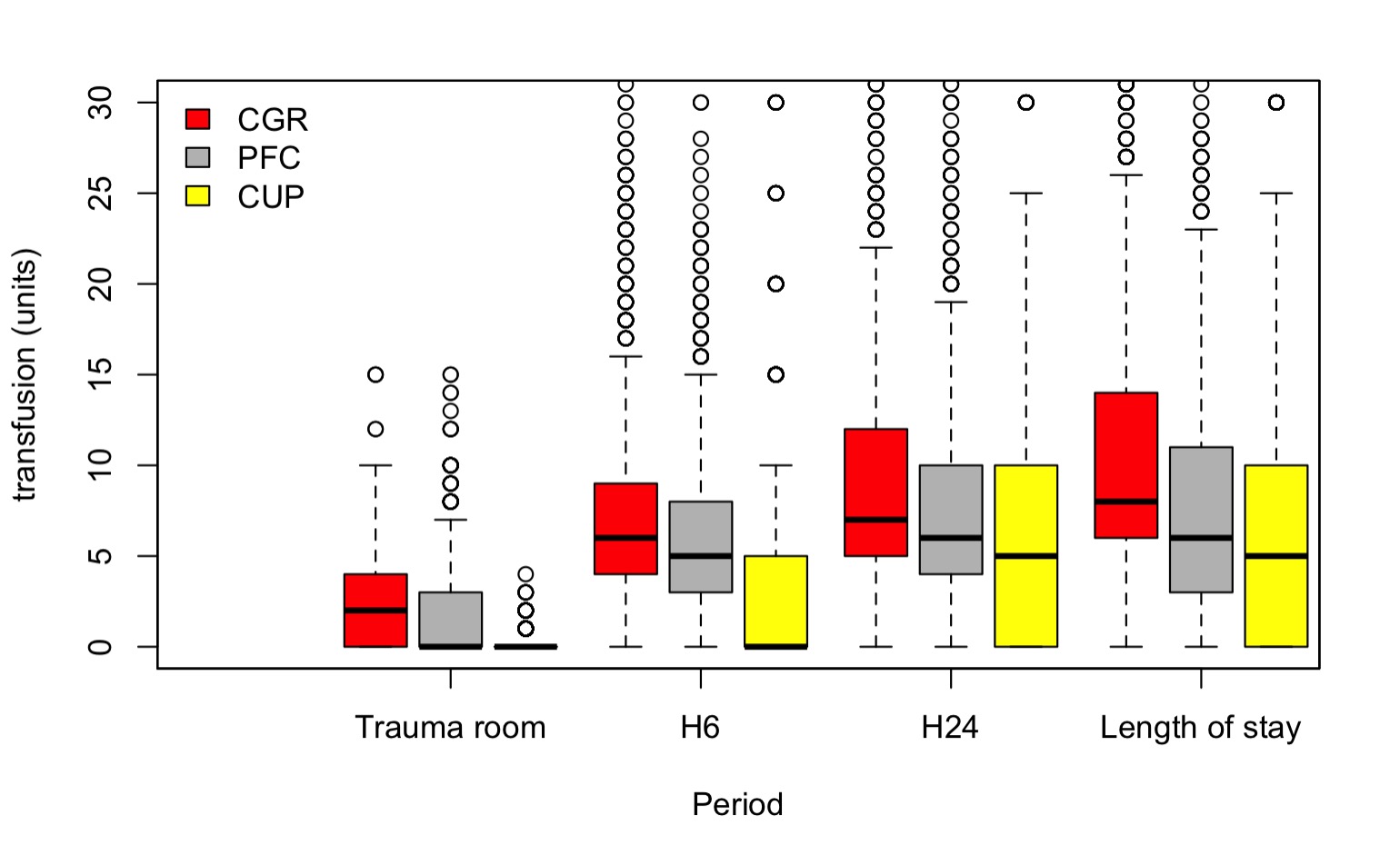

Supplement: Supplementary file 3 — Additional file 3. Fig. S1: Cumulative transfusions of blood products (RBC, FFP, PU) during the trauma patient clinical pathway until ICU discharge [file 13054_2022_3928_MOESM3_ESM.jpg]
